# Correlates of Skeletal Muscle Mass and Differences Between Novel Subtypes in Recent-Onset Diabetes

**DOI:** 10.1210/clinem/dgad605

**Published:** 2023-10-13

**Authors:** Christian Herder, Haifa Maalmi, Nina Saatmann, Oana-Patricia Zaharia, Klaus Strassburger, Volker Burkart, Kristina Norman, Michael Roden

**Affiliations:** Institute for Clinical Diabetology, German Diabetes Center, Leibniz Center for Diabetes Research at Heinrich Heine University Düsseldorf, Düsseldorf 40225, Germany; German Center for Diabetes Research (DZD), Partner Düsseldorf, München-Neuherberg 85764, Germany; Department of Endocrinology and Diabetology, Medical Faculty and University Hospital Düsseldorf, Heinrich Heine University Düsseldorf, Düsseldorf 40225, Germany; Institute for Clinical Diabetology, German Diabetes Center, Leibniz Center for Diabetes Research at Heinrich Heine University Düsseldorf, Düsseldorf 40225, Germany; German Center for Diabetes Research (DZD), Partner Düsseldorf, München-Neuherberg 85764, Germany; Institute for Clinical Diabetology, German Diabetes Center, Leibniz Center for Diabetes Research at Heinrich Heine University Düsseldorf, Düsseldorf 40225, Germany; German Center for Diabetes Research (DZD), Partner Düsseldorf, München-Neuherberg 85764, Germany; Institute for Clinical Diabetology, German Diabetes Center, Leibniz Center for Diabetes Research at Heinrich Heine University Düsseldorf, Düsseldorf 40225, Germany; German Center for Diabetes Research (DZD), Partner Düsseldorf, München-Neuherberg 85764, Germany; Department of Endocrinology and Diabetology, Medical Faculty and University Hospital Düsseldorf, Heinrich Heine University Düsseldorf, Düsseldorf 40225, Germany; German Center for Diabetes Research (DZD), Partner Düsseldorf, München-Neuherberg 85764, Germany; Institute for Biometrics and Epidemiology, German Diabetes Center, Leibniz Center for Diabetes Research at Heinrich Heine University Düsseldorf, Düsseldorf 40225, Germany; Institute for Clinical Diabetology, German Diabetes Center, Leibniz Center for Diabetes Research at Heinrich Heine University Düsseldorf, Düsseldorf 40225, Germany; German Center for Diabetes Research (DZD), Partner Düsseldorf, München-Neuherberg 85764, Germany; Department of Geriatrics and Medical Gerontology, Charité–Universitätsmedizin Berlin, Corporate Member of Freie Universität Berlin and Humboldt-Universität zu Berlin, Berlin 13347, Germany; Department of Nutrition and Gerontology, German Institute of Human Nutrition Potsdam-Rehbrücke, Nuthetal 14558, Germany; Institute of Nutritional Science, University of Potsdam, Nuthetal 14558, Germany; German Center for Cardiovascular Research (DZHK), Partner Site Berlin, Berlin 10785, Germany; Institute for Clinical Diabetology, German Diabetes Center, Leibniz Center for Diabetes Research at Heinrich Heine University Düsseldorf, Düsseldorf 40225, Germany; German Center for Diabetes Research (DZD), Partner Düsseldorf, München-Neuherberg 85764, Germany; Department of Endocrinology and Diabetology, Medical Faculty and University Hospital Düsseldorf, Heinrich Heine University Düsseldorf, Düsseldorf 40225, Germany

**Keywords:** muscle mass, inflammation, diabetes subtypes, classification

## Abstract

**Context:**

Low skeletal muscle mass (SMM) is associated with long-standing diabetes but little is known about SMM in newly diagnosed diabetes.

**Objective:**

We aimed to identify correlates of SMM in recent-onset diabetes and to compare SMM between novel diabetes subtypes.

**Methods:**

SMM was normalized to body mass index (SMM/BMI) in 842 participants with known diabetes duration of less than 1 year from the German Diabetes Study (GDS). Cross-sectional associations between clinical variables, 79 biomarkers of inflammation, and SMM/BMI were assessed, and differences in SMM/BMI between novel diabetes subtypes were analyzed with different degrees of adjustment for confounders.

**Results:**

Male sex and physical activity were positively associated with SMM/BMI, whereas associations of age, BMI, glycated hemoglobin A_1c_, homeostatic model assessment for β-cell function, and estimated glomerular filtration rate with SMM/BMI were inverse (all *P* < .05; model *r*^2^ = 0.82). Twenty-three biomarkers of inflammation showed correlations with SMM/BMI after adjustment for sex and multiple testing (all *P* < .0006), but BMI largely explained these correlations. In a sex-adjusted analysis, individuals with severe autoimmune diabetes had a higher SMM/BMI whereas individuals with severe insulin-resistant diabetes and mild obesity-related diabetes had a lower SMM/BMI than all other subtypes combined. However, differences were attenuated after adjustment for the clustering variables.

**Conclusion:**

SMM/BMI differs between diabetes subtypes and may contribute to subtype differences in disease progression. Of note, clinical variables rather than biomarkers of inflammation explain most of the variation in SMM/BMI.

Skeletal muscle is the main contributor to insulin-mediated glucose disposal (1, 2). Numerous studies have shown an inverse association between skeletal muscle mass (SMM) and type 2 diabetes in observational studies (3). Individuals with more advanced stages of SMM loss (sarcopenia) have a poor long-term prognosis due to a higher risk of morbidity and mortality in acute and chronic diseases (4, 5). A recent mendelian randomization study based on UK Biobank data suggested a causal link between lower muscle mass and higher risk of diabetes (6). Thus, maintaining SMM with increasing age may help prevent the development and progression of diabetes.

Extrinsic contributors to reduced SMM are multifactorial, ranging from low physical activity and inadequate nutrition to polymedication (4, 7-10). Intrinsic factors are still poorly understood. While aging-related processes may partly contribute to reduced SMM in older people, diabetes also accelerates the loss of SMM so that both variables appear interconnected in a bidirectional manner (2, 6). Subclinical inflammation may integrate several of the aforementioned extrinsic and intrinsic risk factors of reduced SMM. Higher circulating levels of proinflammatory biomarkers are associated with loss of SMM (11). Inflammation, SMM, insulin resistance, and diabetes have been proposed to be linked through multiple mechanisms (4, 12), with most evidence coming from studies in geriatric people with sarcopenia and diabetes (13, 14). In contrast, clinical and inflammation-related correlates of SMM in younger people with newly diagnosed diabetes have not yet been studied. Although it is not known to what extent low SMM is a clinically important problem in this group, it is noteworthy that recent studies have provided increasing evidence that many complications and comorbidities usually associated with long-term diabetes are already present shortly after the diagnosis of diabetes (15, 16). Thus, it should be of interest to extend studies on SMM to younger individuals at earlier stages of diabetes.

Recent studies addressing the heterogeneity of diabetes have suggested that stratification of people with diabetes based on 6 clinical clustering variables into 5 novel diabetes subtypes with differences in metabolic and proinflammatory profiles may help improve our understanding of why people differ in the progression of the disease and the risk of complications (17-23). Moreover, it has been shown recently that these subtypes also show differences in cardiorespiratory fitness (24) but it is still unknown whether they also differ in SMM and to what extent differences may be explained by clustering variables or inflammation.

Therefore, this study aims (i) to identify clinical correlates of SMM, normalized to BMI (SMM/BMI) (25-28), in people with recent-onset diabetes in a cross-sectional analysis of baseline data from the German Diabetes Study (GDS) (15), (ii) to characterize inflammation-related correlates of the SMM/BMI ratio using a multimarker panel of biomarkers of inflammation (20), and (iii) to investigate whether people in the novel diabetes subtypes (17, 23) differ in the SMM/BMI ratio and whether clustering variables could explain these differences.

## Materials and Methods

### Study Design and Study Population

This study is based on the GDS (15), an ongoing prospective cohort study that investigates the natural history of recent-onset diabetes and the development of diabetes-associated complications. The GDS enrolls people with recent-onset diabetes (known diabetes duration ≤1 year) and glucose-tolerant individuals serving as a control group, all aged 18 to 69 years. Diagnosis of diabetes was made by the participants’ physicians based on symptoms, during examinations for other reasons or regular health checkups. Diabetes diagnosis was then validated according to the American Diabetes Association criteria at study entry (29). Study design, cohort profile, inclusion, and exclusion criteria have been described previously (15).

The GDS is conducted according to the Declaration of Helsinki, approved by the ethics committee of Heinrich-Heine-University, Düsseldorf, Germany (ref. 4508), and was registered with ClinicalTrials.gov, registration number NCT01055093. All participants provided written informed consent.

As shown in Supplementary Fig. S1 (30), this substudy included 848 consecutive participants with diabetes from the GDS baseline examination with diabetes subtype allocation according to Ahlqvist et al (17). Six participants were excluded because of missing data for the SMM/BMI ratio, leaving a sample of 842 individuals. Out of those, 427 participants had complete data for 7 inflammation-related biomarkers mainly measured by enzyme-linked immunosorbent assay (ELISA) (31, 32), and 490 had complete data for 74 biomarkers of inflammation measured by proximity extension assay technology (20, 33) with an overlap of 371 individuals who had complete data for both biomarker sets.

### Anthropometric Measurements

Body size and weight were measured using a calibrated scale with a stadiometer (SECA674) without shoes and in light underwear to the nearest 0.5 kg and 0.1 cm, respectively (15). BMI was calculated as weight [kg]/(height [m])^2^. Body composition was assessed by bioelectrical impedance analysis (BIA; BioElectrical Impedance Analyzer System, RJL Systems). To avoid any bias due to meal intake and circadian rhythm, the BIA assessment was always performed with fasting study participants between 7 Am and 10 Am. SMM was estimated using the equation by Janssen et al using BIA data (34). This equation was validated against magnetic resonance imaging (MRI) in an adult population varying in age and adiposity (34). Given the correlation between SMM and body size, different standardization methods have been proposed including normalization to BMI (25-28). We followed the approach suggested by the FNIH (Foundation for the National Institutes of Health) Sarcopenia Project group (35) and standardized SMM by calculating the ratio between SMM and BMI (SMM/BMI) for a clinically relevant estimation of muscle mass. Of note, normalization to BMI has been validated by studies showing more pronounced associations of BMI-normalized muscle mass indices with muscle function, cardiometabolic risk factors, and mortality compared to other muscle mass indices (36-38).

### Assessment of Clinical, Metabolic, and Lifestyle Variables

Data for age, sex, time since diabetes diagnosis, lifestyle factors, physical activity, medical history, and medication use were obtained by questionnaires. Hypertension was defined as systolic blood pressure greater than or equal to 140 mm Hg, diastolic blood pressure greater than or equal to 90 mm Hg, or use of antihypertensive medication. Study participants underwent metabolic phenotyping after fasting overnight (≥8 hours) and after having stopped oral glucose-lowering medication for 3 days. Individuals on insulin treatment applied their last insulin dose in the evening before the first examination day. Fasting glucose, fasting C-peptide, glycated hemoglobin A_1c_ (HbA_1c_), glutamic acid decarboxylase antibodies (GADA), and serum lipids were measured as described previously (15, 18). HOMA2 estimates of insulin resistance (HOMA2-IR) and β-cell function (HOMA2-B) were calculated using fasting glucose and C-peptide values (https://www.rdm.ox.ac.uk/about/our-clinical-facilities-and-mrc-units/DTU/software/homa). Estimated glomerular filtration rate (eGFR) was calculated using the CKD-EPI (Chronic Kidney Disease–Epidemiology Collaboration) equation based on serum creatinine and cystatin C (33). Cardiovascular disease (CVD) was defined as self-reported history of myocardial infarction, peripheral arterial occlusive disease, cerebrovascular disease, or stroke.

### Measurement of Biomarkers of Inflammation

This study used 2 sets of biomarkers measured in serum obtained from fasting participants at the baseline examination. The first set of biomarkers included 6 biomarkers measured by ELISA (interleukin [IL]-6, IL-18, soluble intercellular adhesion molecule-1 [sICAM-1], soluble E-selectin [sE-selectin], and total and high-molecular-weight [HMW] adiponectin) and high-sensitivity C-reactive protein (hs-CRP) measured on a Roche/Hitachi c 311 analyzer as described previously (31, 32). The following ELISA kits were used: Human IL-6 Quantikine HS ELISA (R&D Systems; RRID:AB_3065113), Human IL-18 ELISA (MBL; RRID:AB_3065114), Human ICAM-1 Quantikine ELISA (R&D Systems; RRID:AB_3065115), Human E-selectin/CD62E Quantikine ELISA (R&D Systems; RRID:AB_3065116), and Adiponectin (Multimeric) EIA (ALPCO; RRID:AB_3065197). The second biomarker set was based on the OLINK Target 96 Inflammation panel from OLINK Proteomics with assay characteristics as reported in detail previously (20, 33). This assay uses proximity extension assay technology to simultaneously measure 92 protein biomarkers including cytokines, chemokines, growth factors and factors involved in acute inflammatory and immune responses, angiogenesis, fibrosis, and endothelial activation, which are summarily referred to as “biomarkers of inflammation” in this manuscript. The assay yields relative quantifications of biomarker levels given as normalized protein expression values, which are comparable in their distribution to log_2_-transformed protein concentrations. Out of these 92 biomarkers, brain-derived neurotrophic factor could not be quantified due to technical reasons. Additionally, 17 biomarkers were excluded because they had more than 25% of values below the limit of detection, leaving 74 biomarkers.

### Statistical Analysis

Data for continuous variables are given as mean (SD) or median (25th-75th percentiles) and as percentages (%) for categorical variables. Normal distribution of variables was inspected visually with histograms and Q-Q plots. Serum levels of IL-6, IL-18, sICAM-1, sE-selectin, total adiponectin, HMW adiponectin, hs-CRP, and triglycerides were log-transformed prior to analysis to approximate normal distribution.

Differences in clinical variables between sex-specific SMM/BMI quintiles or between diabetes subtypes were assessed by the nonparametric Kruskal-Wallis test, analysis of variance, χ^2^ test, or Cochran-Armitage test for trend.

Associations between clinical variables and the SMM/BMI ratio were assessed using multiple linear regression analysis with all clinical variables entering the model simultaneously. The clinical variables that were investigated consisted of the clustering variables on which the novel subtypes are based (17, 18) and other variables that are readily available in clinical practice.

Associations between biomarkers of inflammation and the SMM/BMI ratio were assessed using partial Spearman correlation coefficients with adjustment for sex (model 1), sex and BMI (model 2), and 7 clinical correlates identified in this study (model 3).

Associations between the novel diabetes subtypes (independent variable) and SMM/BMI (continuous dependent variable) were assessed using generalized mixed models with PROC GLIMMIX. Model 1 was adjusted for sex. Model 2 was additionally adjusted for the clustering variables underlying the diabetes subtypes (age at diagnosis, BMI, HbA_1c_, HOMA2-B, HOMA2-IR, GADA; all covariables used as continuous variables). Model 3 was instead adjusted for the 7 correlates of SMM/BMI identified in this study (sex, age, BMI, eGFR, HOMA2-B, HbA_1c_, physical activity). First, each diabetes subtype was tested against the other subtypes combined as a reference group. Second, the 5 subtypes were tested in pairs (10 pairwise associations) with correction for multiple group comparisons using the Tukey-Kramer test. Associations were estimated with regression coefficients (β; reflecting mean adjusted differences between groups) and their corresponding SEs.

Analyses of clinical correlates, inflammation-related biomarkers, and subtypes were independent from each other, and each analysis was conducted as a complete case analysis without missing values.

All statistical analyses were carried out with SAS version 9.4 (SAS Institute), and *P* values less than .05 were considered as indicators of a statistically significant difference or association unless specified otherwise. In analyses on biomarkers of inflammation, Bonferroni correction for multiple testing was performed (α = .05/79 = 0.0006).

## Results

### Clinical Correlates of the Skeletal Muscle Mass/Body Mass Index Ratio

The distribution of the SMM/BMI ratio stratified by sex is shown in Supplementary Fig. S2 (30). The SMM/BMI ratio was higher in men than in women, so sex-specific quintiles were used for an initial assessment of associations between the SMM/BMI ratio and clinical characteristics of the study population (Table 1). Higher SMM/BMI was associated with younger age, lower BMI, lower β-cell capacity measured with HOMA2-B, higher insulin sensitivity measured with HOMA2-IR, lower levels of triglycerides, lower levels of total cholesterol, higher eGFR, higher prevalence of GADA, higher physical activity, lower prevalence of hypertension and CVD, less frequent use of metformin, more frequent use of insulin, and less frequent use of nonsteroidal anti-inflammatory drugs (NSAIDs) and lipid-lowering drugs. The prevalence of severe autoimmune diabetes (SAID) prevalence was higher with higher SMM/BMI but this was not the case for severe insulin-deficient diabetes (SIDD). Severe insulin-resistant diabetes (SIRD) and mild obesity-related diabetes (MOD) prevalence decreased with higher SMM/BMI. Mild age-related diabetes (MARD) prevalence showed a U-shaped association with highest prevalence levels in SMM/BMI quintiles 2 to 4 (see Table 1).

**Table 1.** Clinical characteristics of the study population stratified by sex-specific quintiles of the skeletal muscle mass/body mass index ratio

		SMM/BMI ratio by sex-specific quintiles*^a^*					
Characteristic	N	Quintile 1 (n = 168)	Quintile 2 (n = 169)	Quintile 3 (n = 168)	Quintile 4 (n = 169)	Quintile 5 (n = 168)	*P^b^*
Age, y	842	55.7 (45.6-62.5)	53.3 (45.8-59.9)	52.8 (45.3-58.7)	47.7 (37.0-55.8)	38.9 (27.3-50.5)	**<.0001**
BMI	842	35.8 (31.6-40.6)	31.5 (28.6-35.6)	29.1 (26.5-32.4)	27.1 (24.8-30.2)	23.7 (21.5-25.4)	**<.0001**
Diabetes subtypes, %	842						**<.0001**
SAID		5%	8%	20%	28%	58%	
SIDD		4%	3%	1%	4%	4%	
SIRD		16%	13%	4%	6%	1%	
MOD		55%	38%	31%	22%	10%	
MARD		20%	38%	44%	40%	27%	
Time since diabetes diagnosis, d	842	162 (92-249)	184 (124; 272)	182 (104; 283)	145 (90; 230)	168 (112; 276)	**.014**
HbA_1c_, mmol/mol	842	46.4 (41.5-54.1)	44.2 (39.9-49.7)	45.3 (41.0-51.9)	44.2 (39.9-50.8)	45.3 (41.0-50.3)	.056
HbA_1c_, %	842	6.4 (6.0-7.1)	6.2 (5.8-6.7)	6.3 (5.9-6.9)	6.2 (5.8-6.8)	6.3 (5.9-6.8)	.056
HOMA2-B	842	100.7 (74.7-132.0)	97.8 (69.9-129.2)	78.9 (61.5-107.5)	72.3 (46.9-105.2)	48.0 (35.9-68.9)	**<.0001**
HOMA2-IR	842	3.1 (2.3-3.7)	2.6 (1.8-3.3)	2.2 (1.5-2.8)	1.7 (1.2-2.6)	1.0 (0.7-1.3)	**<.0001**
GADA > 0.9 units/mL, %	842	5%	8%	20%	28%	58%	**<.0001**
Current smokers, %	842	21%	22%	18%	23%	15%	.173
Physically active, %	836	51%	60%	77%	71%	85%	**<.0001**
eGFR, mL/min per 1.73 m^2^	785	86.0 (75.0-99.2)	89.1 (76.4-101.9)	90.9 (80.6-100.2)	92.4 (81.7-104.1)	98.2 (87.5-108.2)	**<.0001**
Triacylglycerol, mmol/L	842	1.65 (1.25-2.27)	1.62 (1.20-2.32)	1.35 (0.98-1.89)	1.19 (0.85-1.82)	0.84 (0.62-1.19)	**<.0001**
Total cholesterol, mmol/L	842	5.19 (4.60-5.90)	5.24 (4.62-5.83)	4.99 (4.33-5.80)	5.04 (4.31-5.91)	4.67 (4.11-5.13)	**<.0001**
Hypertension, %	834	75%	72%	65%	51%	35%	**<.0001**
CVD, %	827	6%	10%	7%	5%	2%	**.033**
Glucose-lowering drugs, %	830						**<.0001**
None		40%	40%	31%	32%	14%	
Metformin		40%	38%	42%	30%	14%	
Insulin		11%	14%	18%	31%	64%	
Other*^c^*		9%	8%	9%	7%	8%	
Lipid-lowering drugs, %	842	14%	22%	15%	12%	5%	**.0007**
NSAIDs, %	842	21%	21%	16%	14%	4%	**<.0001**

Continuous variables are given as median (25th percentile-75th percentile) and categorical variables are given as percentages (%). *P* values in bold indicate statistical significance at α = .05.

Abbreviations: BMI, body mass index; CVD, cardiovascular diseases; eGFR, estimated glomerular filtration rate; GADA, glutamic acid decarboxylase antibodies; HbA_1c_, glycated hemoglobin A_1c_; HDL, high-density lipoprotein; HOMA2-B, homeostatic model assessment for β-cell function; HOMA2-IR, homeostatic model assessment for insulin resistance; LDL, low-density lipoprotein; MARD, mild age-related diabetes; MOD, mild obesity-related diabetes; NSAIDs, nonsteroidal anti-inflammatory drugs; SAID, severe autoimmune diabetes; SIDD, severe insulin-deficient diabetes, SIRD, severe insulin-resistant diabetes; SMM, skeletal muscle mass.

^*a*^Sex-specific cutoffs between quintiles of SMM/BMI ratio were 1.06, 1.17, 1.26, and 1.40 in men and 0.66, 0.73, 0.81, and 0.93 in women.

^*b*^ *P* value for comparison between sex-specific quintiles of SMM/BMI ratio, by the nonparametric Kruskal-Wallis test, χ^2^ test, or Cochran-Armitage test for trend as appropriate.

^*c*^This category includes sulfonylureas, dipeptidyl peptidase 4 inhibitors, and thiazolidinediones; none of the study participants used glucagon-like peptide-1 receptor agonists or sodium-glucose cotransporter 2 inhibitors.

To identify independent correlates of the SMM/BMI ratio, a multivariable model was calculated. In this model, male sex and physical activity were positively associated with the SMM/BMI ratio, whereas age, BMI, HbA_1c_, HOMA2-B, and eGFR showed inverse associations (Table 2). After the exclusion of variables that did not show statistically significant associations with the SMM/BMI ratio, a more parsimonious model limited to these 7 variables resulted in a coefficient of determination of *r*^2^ = 0.82 (Supplementary Table S1 (30)), indicating that 82% of the SMM/BMI ratio variance can be explained by the variance of the clinical correlates in the model.

**Table 2.** Clinical correlates of the skeletal muscle mass/body mass index ratio

Variable	β	SE	*P*
Sex, male vs female	0.432	9.6E-3	**<.0001**
Age, y	−4.2E-3	5.3E-4	**<.0001**
BMI	−1.6E-2	9.1E-4	**<.0001**
Time since diabetes diagnosis, d	1.7E-5	4.6E-5	.714
HbA_1c_, mmol/mol	−9.9E-4	5.0E-4	**.0493**
HOMA2-B	−4.7E-4	1.4E-4	**.0006**
HOMA2-IR	9.8E-4	1.8E-3	.577
GADA > 0.9 units/mL, %	0.010	0.013	.455
Current smokers, %^a^			
Yes	−1.3E-3	0.014	.929
No	7.9E-3	0.012	.507
Missing	Ref.	Ref.	Ref.
Physically active, %	0.046	9.8E-3	**<.0001**
eGFR, mL/min per 1.73 m^2^	−1.2E-3	3.5E-4	**.0005**
Triglycerides, mmol/L	−1.6E-2	9.7E-3	.099
Total cholesterol, mmol/L	−3.6E-3	4.9E-3	.465
Hypertension, %	−1.2E-2	1.0E-2	.231
CVD, %	−6.2E-2	2.0E-2	.757
Glucose-lowering drugs, %			
None	Ref.	Ref.	Ref.
Metformin	1.4E-2	1.1E-2	.213
Insulin	1.0E-2	1.5E-2	.474
Other	2.3E-2	1.7E-2	.189
Lipid-lowering drugs, %	9.8E-3	1.4E-2	.488
NSAIDs, %	−4.4E-3	1.3E-2	.745

The regression model included all variables from Table 1 (excluding subtypes and HbA_1c_, %) and was based on a complete-case analysis (n = 750). Triglycerides were log-transformed before entering the model. *R*^2^ = 0.824.

Abbreviations: BMI, body mass index; CVD, cardiovascular diseases; eGFR, estimated glomerular filtration rate; GADA, glutamic acid decarboxylase antibodies; HbA_1c_, glycated hemoglobin A_1c_; HOMA2-B, homeostatic model assessment for β-cell function; HOMA-IR, homeostatic model assessment for insulin resistance; NSAIDs, nonsteroidal anti-inflammatory drugs; Ref., reference; SMM, skeletal muscle mass.

^*a*^This variable was coded in 3 categories: yes (current smokers), no, and missing (no information on smoking status).

### Skeletal Muscle Mass/Body Mass Index Ratio and Biomarkers of Inflammation

Mean serum levels of 79 biomarkers of inflammation stratified by sex are shown in Supplementary Table S2 (30). Differences between groups at *P* less than .05 were observed for 34 biomarkers, and between-group differences for 19 biomarkers remained statistically significant after adjustment for multiple testing. To assess the extent to which the associations between biomarkers of inflammation and the SMM/BMI ratio were independent of confounders, partial correlation coefficients were calculated (Supplementary Table S3 (30)). In model 1, 19 biomarkers showed a significant inverse correlation (*r* = −0.467 to −0.184) after adjustment for sex and multiple testing, whereas 4 biomarkers showed a positive correlation (*r* = 0.198-0.274) (Table 3). Given the strong correlations of BMI with the SMM/BMI ratio and subclinical inflammation, the effect of BMI as a confounder was assessed separately. After additional adjustment for BMI (model 2), all correlations were attenuated and statistical significance was lost. Further adjustment for the remaining clinical correlates of the SMM/BMI ratio (ie, age, HbA_1c_, HOMA-2B, physical activity, and eGFR; model 3) had almost no effect on correlation coefficients.

**Table 3.** Correlations between biomarkers of inflammation and skeletal muscle mass/body mass index ratio

	Model 1		Model 2		Model 3	
Biomarker	*r*	*P*	*r*	*P*	*r*	*P*
4E-BP1	−0.190	.0002	−0.050	.348	−0.049	.356
Adiponectin-HMW, ng/mL	0.198	.0001	−0.008	.884	−0.011	.839
Adiponectin-total, ng/mL	0.238	<.0001	0.016	.761	0.014	.797
CCL19	−0.257	<.0001	−0.089	.096	−0.088	.100
CCL20	−0.184	.0004	−0.132	.013	−0.131	.014
CCL3	−0.221	<.0001	−0.034	.523	−0.034	.526
CD40	−0.194	.0002	−0.099	.063	−0.098	.066
CDCP1	−0.390	<.0001	−0.037	.482	−0.036	.503
CSF-1	−0.199	.0001	−0.108	.041	−0.108	.043
FGF-21	−0.370	<.0001	−0.009	.859	−0.008	.877
Flt3L	−0.245	<.0001	−0.047	.381	−0.046	.394
HGF	−0.354	<.0001	−0.059	.265	−0.058	.277
hs-CRP, mg/L	−0.442	<.0001	−0.097	.068	−0.097	.068
IL-18R1	−0.230	<.0001	−0.089	.095	−0.089	.097
IL18	−0.225	<.0001	−0.063	.240	−0.062	.245
IL6	−0.467	<.0001	−0.091	.088	−0.091	.089
MCP-3	−0.329	<.0001	−0.109	.040	−0.108	.042
OPG	−0.256	<.0001	−0.039	.468	−0.037	.489
SCF	0.274	<.0001	0.060	.256	0.059	.268
sE-Selectin, ng/mL	−0.263	<.0001	−0.040	.452	−0.038	.472
sICAM1, ng/mL	−0.217	<.0001	−0.105	.048	−0.105	.048
SLAMF1	−0.184	.0004	−0.030	.571	−0.029	.583
TWEAK	0.221	<.0001	0.016	.763	0.015	.773

This table summarizes 23 biomarkers with statistically significant associations at *P* < .0006 in model 1. The full list of results for all 79 biomarkers is given in Supplementary Table S2 (30). Biomarker units are normalized protein expression if not indicated otherwise.

Model 1: partial correlation adjusted for sex (n = 371).

Model 2: partial correlation adjusted for sex and BMI (n = 371).

Model 3: partial correlation adjusted for 7 clinical correlates (n = 360).

Abbreviations: BMI, body mass index; FGF-21, fibroblast growth factor-21; HMW, high-molecular-weight; hs-CRP, high-sensitivity C-reactive protein; IL, interleukin; SCF, stem cell factor; SMM, skeletal muscle mass; TWEAK, TNFSF12/tumor necrosis factor ligand superfamily member 12.

### Skeletal Muscle Mass/Body Mass Index Ratio and Diabetes Subtypes

**Figure 1.**
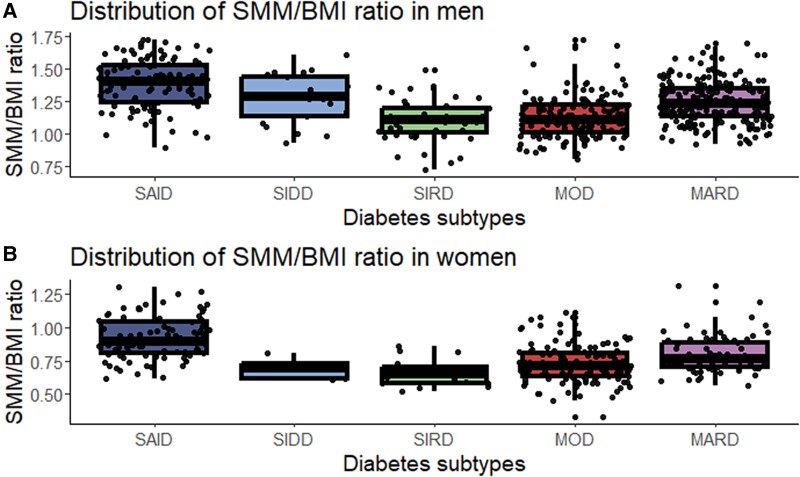
Distribution of SMM/BMI ratio according to sex and diabetes subtypes. The distribution of the SMM/BMI ratio is given stratified by sex (A, men; B, women). Box plots show median values with 25th and 75th percentiles. The upper boxplot whiskers represent the maximum value or the last data point within the range of 75% quantile + 1.5 IQR; lower boxplot whiskers represent the minimum value or the last data point within the range of 25% quantile −1.5 IQR.

The SMM/BMI ratio differed across diabetes subtypes both in men and women and was lowest in people with SIRD and highest in people with SAID (Fig. 1; Supplementary Table S4 (30)). Detailed clinical characteristics of the individuals in the 5 subtypes are shown in Supplementary Table S5 (30).

In a sex-adjusted analysis, individuals with SAID had a higher SMM/BMI ratio, and individuals with SIRD and MOD had a lower SMM/BMI ratio compared to all other subtypes combined (Table 4; model 1). Given that age and BMI are closely associated with both SMM/BMI and the diabetes subtypes, we calculated 2 additional models to assess to what extent these differences persist when taking these and other variables into account. After adjustment for sex and the clustering variables, only the positive association between SAID and the SMM/BMI ratio remained significant (model 2). In contrast, adjustment for the 7 clinical correlates identified in this study revealed a lower SMM/BMI ratio in the subtype with SIDD compared to all other subtypes combined (model 3). These findings suggest that subtype differences are partially explained by anthropometric and metabolic confounders.

**Table 4.** Associations between each diabetes subtype and skeletal muscle mass/body mass index ratio

	Model 1 n = 842			Model 2 n = 842			Model 3 n = 780		
Subtype comparisons	β	SE	*P*	β	SE	*P*	β	SE	*P*
SAID vs all others	**0.188**	**0.013**	**<.0001**	**0.040**	**0.014**	**.005**	0.015	0.012	.216
SIDD vs all others	0.010	0.036	.775	−0.054	0.030	.071	**−0.063**	**0.031**	**.043**
SIRD vs all others	**−0.143**	**0.019**	**<.0001**	0.020	0.019	.290	0.025	0.020	.214
MOD vs all others	**−0.127**	**0.012**	**<.0001**	−0.019	0.011	.091	−0.010	0.011	.330
MARD vs all others	0.013	0.012	.305	−0.009	0.012	.454	−0.003	0.012	.780

Model 1: adjusted for sex.

Model 2: adjusted for sex + age, BMI, HbA_1c_, HOMA2-B and HOMA2-IR, and GADA.

Model 3: adjusted sex + age, BMI, eGFR, HOMA2-B, HbA_1c_, and physical activity.

Bold print indicates statistical significance at *P* less than .05.

Abbreviations: BMI, body mass index; eGFR, estimated glomerular filtration rate; GADA, glutamic acid decarboxylase antibodies; HbA_1c_, glycated hemoglobin A_1c_; HOMA2-B, homeostatic model assessment for β-cell function; HOMA2-IR, homeostatic model assessment for insulin resistance; MARD, mild age-related diabetes; MOD, mild obesity-related diabetes; SAID, severe autoimmune diabetes; SIDD, severe insulin-deficient diabetes; SIRD, severe insulin-resistant diabetes.

In pairwise analyses between individual subtypes, the SMM/BMI ratio was higher in SAID than in all other subtypes, lower in SIRD vs SIDD and MARD, and lower in MOD vs MARD after adjustment for sex (Supplementary Table S6; model 1 (30)). However, none of these associations remained statistically significant after further adjustment in models 2 and 3.

## Discussion

This study demonstrates that (i) age, sex, physical activity, BMI, HbA_1c_, β-cell function, and eGFR explained most of the variance of the SMM/BMI ratio in recent-onset diabetes; (ii) multiple biomarkers of inflammation were correlated with SMM/BMI after adjustment for sex and multiple testing, but these correlations were lost after adjustment for the other correlates; and that (iii) the novel diabetes subtypes differ in the SMM/BMI ratio.

### Clinical Correlates of the Skeletal Muscle Mass/Body Mass Index Ratio

This study identified male sex and physical activity as positive correlates and age, BMI, HbA_1c_, β-cell function, and eGFR as negative correlates of the SMM/BMI ratio, explaining more than 80% of its variance in people with recent-onset diabetes. The findings extend previous analyses because comparable studies including participants with recent-onset diabetes are not available. When we assessed correlates of the SMM/BMI ratio, we focused on variables that are easily available in clinical practice, such as BMI as an accepted surrogate of obesity. Although the average duration since the time of diagnosis was less than 6 months, initial changes in the clinical variables due to diagnosis-related changes in medication and lifestyle cannot be excluded.

Our data on higher SMM in men compared to women align with previous findings from other cohorts (39). A recent meta-analysis on older adults found a positive association of low SMM with age and an inverse association with physical activity in community-based older adults (40), which our study confirms. Studies in people with long-standing type 2 diabetes reported that physical activity is associated with lower odds of sarcopenia, whereas higher age, higher HbA_1c_, higher body fat, and diabetic nephropathy are associated with higher odds of sarcopenia (10, 41). Although the extent of adjustment for confounders and the heterogeneity between studies differed widely, the data appear in line with our study showing that people with higher SMM/BMI are younger, leaner, more insulin-sensitive, and have lower serum lipids and higher eGFR. This suggests that the aforementioned associations may not depend on the time since diabetes diagnosis or the extent of loss of SMM. Of note, our analyses were based on the known diabetes duration, that is, time since clinical diabetes diagnosis. Particularly, participants in subtypes reflecting mainly type 2 diabetes (SIDD, SIRD, MOD, MARD) likely had a history of longer-term undetected hyperglycemia, which could have affected SMM. However, it should be noted that an inverse association between HbA_1c_ and the skeletal muscle index was also found in people without diabetes (39). In our study, this inverse association between SMM/BMI and HbA_1c_ was weak and became statistically significant only in the multivariable model.

We observed an inverse association of HOMA2-B with the SMM/BMI ratio, but not for HOMA2-IR, in the multivariable model, that is, after adjustment for age, BMI, and other relevant confounders. Data from the Third National Health and Nutrition Examination Survey showed an inverse association of HOMA-IR (along with HbA_1c_ and the prevalence of prediabetes) with the skeletal muscle index in a nondiabetic cohort (39). Both HOMA2 indices are related, and higher β-cell function defined using HOMA2-B most likely reflects higher insulin demand because of higher insulin resistance. We used these easily available parameters in our analysis because they were also components of the clustering algorithm defining the novel diabetes subtypes (17), which were analyzed in the context of muscle mass in this study for the first time. Nevertheless, the relative contributions of insulin resistance and β-cell function to muscle mass loss in people with diabetes need to be further investigated by more sophisticated measures of insulin sensitivity and secretion as well as in prospective studies to assess temporal and potential causal relationships.

Of note, study participants were asked to take the last insulin dose in the evening before the baseline examination and to drop other glucose-lowering drugs 3 days before to minimize interference with clinical measurements on the examination days. Most study participants in this sample had measurable C-peptide levels, arguing for residual β-cell function also in people with autoimmune diabetes. This is in line with previous studies that have shown that shortly after diagnosis, individuals with autoimmune diabetes feature partly preserved β-cell function and may not require exogenous insulin treatment, reflecting the so-called “honeymoon phase” (41).

It is possible that a multivariable model containing variables such as dynamic measures of insulin sensitivity and secretion could explain an even larger part of the variance of the SMM/BMI ratio. The assessment of the disposition index could help to clarify to what extent the association with insulin secretion is independent of insulin resistance. Further improvements could be possible by using objective measurements of physical activity and physical fitness.

Furthermore, this study demonstrated that 7 clinical variables explained more than 80% of the variance in the SMM/BMI ratio. Prospective studies would also be relevant to assess to what degree these variables could identify people with recent-onset diabetes who are at higher risk of loss of muscle mass with increasing duration of diabetes. This would be important for better stratified recommendations to perform or increase resistance exercise as the best way to maintain muscle mass, although nutritional interventions may confer small additional benefits (2, 42-45).

### Skeletal Muscle Mass/Body Mass Index Ratio and Biomarkers of Inflammation

This study identified multiple biomarkers of inflammation that were—mostly inversely—correlated with SMM/BMI in people with recent-onset diabetes, which has not been reported before.

Associations between inflammation and loss of muscle mass and/or function have mainly been investigated in the context of aging, type 2 diabetes, and sarcopenic obesity, which are all characterized by increases in circulating levels of proinflammatory cytokines and other proteins (12). This is paralleled by the activation of proinflammatory pathways in the skeletal muscle, immune cell infiltration, exacerbated insulin resistance, disturbed protein synthesis, and increased protein catabolism (12, 46). The list of biomarkers with inverse correlations with SMM/BMI also includes myokines that are released by skeletal muscle such as IL-6 and fibroblast growth factor-21 (46). Thus, our findings are biologically plausible.

Of note, we found positive correlations with SMM/BMI for adiponectin (total adiponectin and the HMW isoform), stem cell factor, and TWEAK (TNFSF12/tumor necrosis factor ligand superfamily member 12). Concentrations of adiponectin, an adipokine with anti-inflammatory, insulin-sensitizing, and promyogenic properties (47, 48), were lower in people with sarcopenia (13), so a positive correlation of serum adiponectin with SMM/BMI is in line with mechanistic and clinical studies. Stem cell factor is a growth factor with multiple functions in hematopoiesis, pigmentation, fertility, gut movement, and neuronal development, whereas its relationship with muscle physiology is less clear (49). In contrast, TWEAK is a cytokine implicated in muscle atrophy, injury, and sarcopenia (13). However, it is still controversial whether these associations primarily reflect degenerative or regenerative effects of TWEAK (50).

An important aspect of this study is the novel finding that the aforementioned associations between biomarkers of inflammation and SMM/BMI in people with recent-onset diabetes are largely explained by BMI and therefore do not represent independent correlates of muscle mass. This result is in line with our previous reports using overlapping study samples that showed that most of the subtype-specific proinflammatory biomarkers in this multimarker panel showed positive correlations with BMI, whereas adiponectin was inversely correlated with waist circumference (20, 32). Studies on sarcopenia in the older general population or in people with long-standing diabetes reported positive associations between proinflammatory biomarkers and cytokines with most consistent associations for hs-CRP (40, 51). However, study designs and selection of confounders in the statistical analyses were heterogeneous, so currently no blood biomarkers for sarcopenia are available that provide additional diagnostic or prognostic benefit beyond routine clinical parameters (4). Importantly, our findings do not preclude that in particular proinflammatory biomarkers may be biologically relevant because they might contribute to the higher cardiometabolic and mortality risk in people with lower compared to those with higher SMM/BMI ratio.

### Skeletal Muscle Mass/Body Mass Index Ratio and Diabetes Subtypes

Another novel observation of this study is the lower SMM/BMI ratio in SIRD and MOD and the higher SMM/BMI ratio in SAID after adjustment for sex. Additional adjustment for the clustering variables underlying the subtype definition (model 2) or for the correlates of SMM/BMI identified in this study (model 3) point toward a higher SMM/BMI ratio in SAID and a lower SMM/BMI ratio in SIDD, respectively, compared to the other subtypes.

The adjustment for BMI may appear surprising given that BMI was used to calculate the SMM/BMI ratio. However, the clustering algorithm for the diabetes subtypes uses BMI at diagnosis rather than waist circumference or other indices of obesity. Since we aimed to test if differences between subtypes are independent of the clustering variables, the adjustment for BMI was an essential part of the analysis plan.

The sex-adjusted differences in the SMM/BMI ratio are in line with a previous study from the GDS that showed similar findings for physical fitness, that is, highest maximal oxygen consumption (VO_2_max) for SAID and lowest VO_2_max for SIRD and MOD (24). However, our study also indicates that confounding factors partially explain the associations between diabetes subtypes and SMM/BMI. Depending on the selection of covariables, SAID showed a higher and SIDD a lower SMM/BMI ratio than the other subtypes. In particular, the analyses for SIDD are limited by the low number of study participants in this subtype, so validation in other cohorts would be desirable.

To assess the clinical implications of our results, longitudinal studies would be beneficial to characterize reductions in SMM/BMI due to decreases in SMM and/or increases in fat mass in the diabetes subtypes during disease progression. One may expect that a more rapid decline in SMM/BMI would be associated with an increased risk of sarcopenia and related complications, such as CVD and frailty in older individuals (10, 40, 52). Thus, monitoring trajectories of SMM/BMI in people with diabetes could help identify people with declining SMM who might benefit from a combination of resistance exercise and nutritional modification (4, 52).

### Strengths and Limitations

A major strength of this study is the unique focus on people with recent-onset diabetes. In contrast, other studies investigating correlates of muscle mass and sarcopenia mainly focused on older people with longer diabetes duration, which is usually linked to (i) longer exposure to chronic hyperglycemia and inflammation, and (ii) the presence of other diabetes-related comorbidities that may have effects on behavioral risk factors and protein metabolism. Moreover, the study cohort was characterized by comprehensive metabolic phenotyping allowing the analysis of the SMM/BMI ratio in the novel subtypes and by extensive immunological phenotyping.

Limitations of our study include the cross-sectional design, which precluded the identification of genuine risk factors of SMM loss, and the calculation of the SMM/BMI ratio based on BIA rather than MRI. However, it needs to be noted that the equation for SMM was validated against MRI as the gold-standard method with a correlation of *r*^2^ = 0.86 between both methods (34). This validation was performed in a study sample with wide age and BMI ranges (18-86 years and 16-48, respectively), thus including the distribution in age and BMI from our cohort. It is unlikely that a recent diagnosis of diabetes would have a notable effect on the validity of our assessment of SMM. Data for skeletal muscle strength or quality and physical performance were not available and could thus not be included in our analyses. We were also unable to analyze whether the SMM/BMI ratio was independently associated with activities of daily living to detect potential impairments of their functional status (not assessed in the study) or with mortality during the follow-up (low number of deaths). In addition, we could not analyze the relevance of dietary factors and total energy intake because of a high proportion of missing values for this study sample. We focused on people with diabetes given the smaller sample size and the unavailability of biomarker measurements for glucose-tolerant people in the GDS, so further, ideally population-based studies should be conducted to compare findings on clinical and inflammation-related correlates with the SMM/BMI ratio between people without and with diabetes. People with malignancies during the last 5 years and clinically apparent endocrine diseases prior to the baseline examinations that could affect SMM were excluded. Of note, there was no screening for specific subclinical endocrine diseases such as hypogonadism. Finally, study participants were mainly of European descent, which may limit the generalizability of our findings to people with diabetes of other ethnicities.

## Conclusions

This study identified age, sex, physical activity, BMI, HbA_1c_, β-cell function, and eGFR as clinical correlates of the SMM/BMI ratio in people with recent-onset diabetes. Multiple biomarkers of inflammation were also associated with the SMM/BMI ratio, but not independently of the other aforementioned correlates of the SMM/BMI ratio. Differences between novel diabetes subtypes in the SMM/BMI ratio suggest that people with SIDD may benefit from resistance exercise and that the SMM/BMI ratio should be considered when discussing preferences for pharmacological treatment.

## GDS Group

The GDS Group consists of M. Roden (speaker), H. Al-Hasani, B. Belgardt, E. Lammert, G. Bönhof, G. Geerling, C. Herder, A. Icks, K. Jandeleit-Dahm, J. Kotzka, O. Kuß, W. Rathmann, S. Schlesinger, V. Schrauwen-Hinderling, J. Szendroedi, S. Trenkamp, R. Wagner, and their colleagues who are responsible for the design and conduct of the GDS.

## Data Availability

The data are subject to national data protection laws. Therefore, data cannot be made freely available in a public repository. However, data can be requested through an individual project agreement with the Steering Committee of the GDS (speaker: Michael Roden, michael.roden@ddz.de).

## Prior Presentation

Parts of this study were presented at the Diabetes Congress of the German Diabetes Association (DDG), Berlin, Germany, May 25-28, 2022.

## Abbreviations

BIA: bioelectrical impedance analysis
BMI: body mass index
CVD: cardiovascular diseases
eGFR: estimated glomerular filtration rate
ELISA: enzyme-linked immunosorbent assay
GADA: glutamic acid decarboxylase antibodies
GDS: German Diabetes Study
HbA_1c_: glycated hemoglobin A_1c_
HMW: high-molecular-weight
HOMA2-B: homeostatic model assessment for β-cell function
HOMA-IR: homeostatic model assessment for insulin resistance
hs-CRP: high-sensitivity C-reactive protein
IL: interleukin
MARD: mild age-related diabetes
MOD: mild obesity-related diabetes
MRI: magnetic resonance imaging
NSAIDs: nonsteroidal anti-inflammatory drugs
SAID: severe autoimmune diabetes
SIDD: severe insulin-deficient diabetes
SIRD: severe insulin-resistant diabetes
SMM: skeletal muscle mass
TWEAK: TNFSF12/tumor necrosis factor ligand superfamily member 12
